# And the Raven, Never Flitting, Still Is Sitting, Still Is Sitting^1^

**DOI:** 10.3201/eid1610.AC1610

**Published:** 2010-10

**Authors:** Polyxeni Potter

**Affiliations:** Author affiliation: Centers for Disease Control and Prevention, Atlanta, Georgia, USA

**Keywords:** Art science connection, emerging infectious diseases, art and medicine, Clive Hicks-Jenkins, figurative painting, The Prophet Fed by a Raven, zoonotic infections, Welsh artists, animal population surveillance, about the cover

**Figure:**
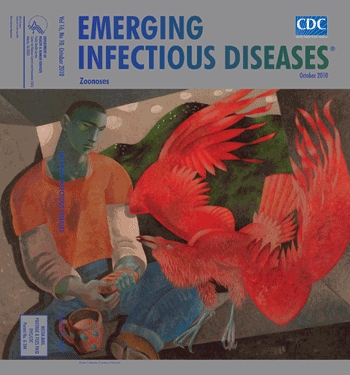
**Clive Hicks-Jenkins (b. 1951), *The Prophet Fed by a Raven* (2007)** Acrylic on panel (62 cm × 82 cm), Courtesy of the artist, private collection, www.hicks-jenkins.com

“At my studio back in Cardiff, the walls swarm with a cast of hermits, angels, penitents, devils, wild beasts, and anchorites,” wrote Clive Hicks-Jenkins during a visit to Prague. “They are made of roughly painted card, jointed for articulation and capable of surprisingly varied and unlikely positions, rather like elaborate shadow puppets. They were constructed as studio aids to achieve a more expressive use of the human figure and free me from the choreographer’s understanding of the body.”

A native of Wales, Hicks-Jenkins spent the first 25 years of his working life as stage designer, choreographer, and theater director. The figures in Gothic Bohemian paintings in the National Gallery in Prague reminded him of the population on his own studio walls. He saw in these figures a kind of “postural distortion,” affirming his notion that “This is not about flesh and the corporeal body.”

He was born in Newport and showed early artistic talent, which he cultivated in London at the Rambert Dance Company and The Italia Conti Academy. He traveled widely in Europe and America, touring with dance and theater troops, but settled back in Wales in the 1980s to focus on his love of painting. His studio, the “battery,” he shares with Pipistrelle bats, which “roost in the roof space above and make frequent forays to pepper my paintings with their droppings. I don’t mind. I enjoy their company.”

Hicks-Jenkins’ painting has been described as figurative, a term now encompassing any form of modern art characterized by references to the real world. “I am not at all captivated by ‘likeness’ or the capturing of it. Most of the figures in my paintings are of ‘types’ rather than specific, identifiable sitters.”He has experimented with various styles, using elements from them in his own work, which is always changing. “I continue to lay myself open to the currents that carry me in new directions.... Every time I reach a point where skill becomes even close to practiced and reliable, I feel the urge to derail the train.”

Hicks-Jenkins’ artistic talent was nourished by his years in the theater. “The stage is a revealing space, much like a painting. All the attention is focused on the limited area, and everything within it becomes more important.” The stage is a rich source of compositional elements. “I’ve developed a technique of making three-dimensional, articulated paper maquettes as part of my preparation for painting at the easel. These fragile little works are pieced together quickly from thin card, cut, and then worked in frottage, monoprint, conté crayon, and acrylic. The puppets are held together with hidden, brass paper fasteners.” He uses maquettes “not as convenient stand-ins for people” but to resolve a common dilemma of the artist pursuing an independent creation, an idea, instead of *mimesis*, an imitation of nature. These puppets, reminiscent of animation figures, distract him from the usual objects of observation, enabling him to concentrate on “Inventing dynamic though often anatomically impossible arrangements of limbs that a live model quite simply couldn’t provide” and to fill space. “They’ve been crucial too in helping me flatten out and pile up shapes…. The paintings become almost like collages.”

The creative effort of imagining and inventing codes and other means of communication goes back to prehistoric times and may be the factor distinguishing humans from other animals. We search for such codes throughout the millennia, trying to decipher human history. Many of the messages we find are from the visual arts, which appeal to the senses as well as the intellect. Mental and visual communications are integrated by details or cues to make characters or situations recognizable without captions. To this end, art often resorts to iconography, a tradition of communicating through images. In this tradition, recurrent figures may be portrayed with the same facial features or clothing, despite changing eras or styles. They may be painted in certain colors or be accompanied by recognized objects or animals. Once the subject becomes identifiable, other elements may be added that elevate the meaning from historical to universal.

Like many of Hicks-Jenkins’ works, *The Prophet Fed by a Raven*, on this month’s cover, contains elements of iconography placed in a contemporary setting and filled with timeless universal themes. The clues point to well-loved prophet Elias (also Elijah) of the Old Testament, an elusive figure of swift foot and obscure origins. The artist placed him on a ledge, as “He dwelled in the clefts of the torrents or in the caves of mountains” and lived the life of “a true son of the desert.” “Nothing was too high for him, and when he was laid on sleep his body prophesied.” He was “as fire, and his word burnt like a torch.” He appeared abruptly on the historical scene, delivered his message against the corruption of his age, lived off charity under conditions of drought and famine, and vanished to the east of the Jordan, where legend has it, ravens “brought him bread and flesh in the evening, and he drank of the torrent.”

Basic elements aside, the artist departs from iconography. The raven is true enough to form in its shaggy throat feathers, wedge-shaped tail, slender figure, narrow wings, and long, thin “fingers” at the wingtips. But it defies traditional color for flaming red and seems far more fiery than the prophet. The remote mountain backdrop is telescoped and manipulated into the top of the composition, placed within reach, virtually inside the window frame. Perched solidly on the edge, the bird turns one sharp eye toward its charge, Bowie knife of a beak parted as if in conversation with the figure slouched enigmatically in the foreground.

The raven, among the most intelligent of birds, is a frequent visitor in the arts where, as in life, it has a contradictory presence, signaling both life and death. Stately and handsome in its shiny uniform coat―black down to the legs, eyes, and beak―it is graceful and inquisitive. Despite its unsavory reputation as an indiscriminate eater, it struts, soars, and glides confidently in virtually all environments and around humans and other animals. “I have no interest in expressions of the stories where the nature of the wild is perverted from its true self,” the artist says about his portrayal of animals. “For me the true miracle of relationships that break the usual mold is that the animal moderates its behavior because it’s moved to.”

In *The Prophet Fed by a Raven*, the bird breaks from ‘likeness’ to become a type, emblematic not only of what can happen between prophets and birds but of all animal–human interactions. In many of these, the roles are often reversed, as in this painting, where the bird takes care of the prophet living in the wild. Sometimes, the raven becomes the prophet, as in the case of West Nile virus spread in North America, when dying birds of the crow family, including ravens, foretold human infection in the New World. But ravens are not alone. With many infections emerging first in animals in remote and underprivileged settings, surveillance of animal health can forecast disease risks to humans. In Sri Lanka, field veterinarians used mobile phones to report animal health information, confirming that this type of animal population surveillance can work in isolated areas with limited resources. While the prophet–raven platform has changed with the times, the universal human–animal interface and its zoonotic consequences, including disease transmission, remain unchanged.
